# Urban Heat Island Mitigation: GIS-Based Analysis for a Tropical City Singapore

**DOI:** 10.3390/ijerph191911917

**Published:** 2022-09-21

**Authors:** Ya Hui Teo, Mohamed Akbar Bin Humayun Makani, Weimeng Wang, Linglan Liu, Jun Hong Yap, Kang Hao Cheong

**Affiliations:** 1 Science, Mathematics and Technology Cluster, Singapore University of Technology and Design, 8 Somapah Road, Singapore S487372, Singapore; 2 Humanities, Arts and Social Sciences Cluster, Singapore University of Technology and Design, 8 Somapah Road, Singapore S487372, Singapore; 3 Information Systems Technology and Design Cluster, Singapore University of Technology and Design, 8 Somapah Road, Singapore S487372, Singapore; 4 Department of Real Estate, National University of Singapore, 4 Architecture Drive, Singapore S117566, Singapore

**Keywords:** urban heat island, energy consumption, Green Mark commercial buildings, sustainable environment, air temperature, greeneries, climate change

## Abstract

To reduce the pace of climate change and achieve the goals set in Paris Agreement by 2030, Association of Southeast Asian Nations (ASEAN) countries have started to prioritize sustainability as one of their top agendas. Numerous studies have demonstrated that one of the most important issues that must be addressed to halt climate change is the urban heat island (UHI). Given the different mitigation strategies available, the focus of our study here is to assess the influence of green spaces and Green Mark commercial buildings on Singapore’s temperature distribution using non-exhaustive factors related to energy consumption and efficiency. Additionally, this paper examines the effectiveness of green spaces and commercial buildings in reducing the rate of temperature change. This study uses ArcGIS software to map data, perform spatial analysis through cloud-based mapping, and produce visual representations with geographic information systems (GIS) to promote greater insight on the formulation of goals and policy making for strategic management. In comparison to non-commercial districts, our findings show that commercial districts have the lowest percentage of temperature change, an estimated 1.6 percent, due to a high concentration of green spaces and Green Mark commercial buildings. Our research also helps to close the research gaps in determining the efficacy of Green Mark commercial buildings, skyrise greeneries, gardens, and national parks. It also helps to minimize the bottleneck of expensive building costs and environmental damage that would have occurred from a design flaw found too late in the urban planning and construction process.

## 1. Introduction

The urban heat island (UHI) is a phenomenon that reflects microclimatic changes due to rapid urbanization [1,2,3,4,5]. As the name suggests, it describes the warmness of both the environment and surfaces of urban areas as compared to non-urbanized areas [6,7,8,9]. There are various factors that cause UHIs, such as having densely populated buildings in an urban area and different types of building materials. Densely built buildings in an urban area could increase thermal capacity and reduce wind speed. This could affect the thermal comfort of the building occupants [10]. Depending on the types of building materials, heat will be trapped during the day and released at night, which increases the diurnal temperature [11]. For instance, buildings with dark exterior surfaces will absorb solar radiation and, in turn, increase cooling load and energy consumption [6,12,13,14]. This does not only apply to buildings, but other man-made constructions such as roads and pavements. Thus, this could be one of the reasons why buildings consume an estimated 30% to 40% energy consumption globally due to an increase in external temperature, which necessitates an increase in cooling load to cool down the internal space [15]. This will also result in an increase in aerial anthropogenic heat emissions in the atmosphere [2,16].

Other factors that affect the UHI include artificial heat produced from industries, vehicles’ combustion gases and air compressors that leads to warmer temperatures. Global temperatures have been gradually increasing, which has amounted to an estimated 1.2 ℃ from 1850 to 2019. This is due to an increase in greenhouse gas emissions which contributes to global warming and climate change [17]. There are some possible factors that contribute to increasing urban temperatures, include the reduction in green space to make way for building new commercial/residential buildings [18].

Heat waves are also a critical factor that could accelerate the process of the UHI [11,19,20,21] through modifying irregular latent and sensible heat [22] as well as affecting wind speed [23]. Positive synergies were found between heat waves and UHI intensities [19,20] as well as daily highest temperature [12,24]. It was found that UHI intensities were generally higher during the heat waves, with an increase of a minimum of 0.4 °C to a maximum of 2 °C in the United States [25,26].

These critical factors that can accelerate the process of the UHI can be further expressed in a surface energy balance equation [27]:

Q* + QF = G + H + LE

where Q* represents net radiation, QF signifies anthropogenic heat, G indicates the storage of heat resulting from the surface’s heat conduction, and H and LE represent sensible and latent heat transfer, respectively.

In general, UHIs can make an impact on urban climate, living environment and energy consumption, which can lead to thermal discomfort, higher temperatures and even air and water pollution. This may speed up urban smog formation if the situation continues to worsen [28,29]. For instance, Shastri et al. [30] studied the surface urban heat island intensity (SUHII) in India and discovered that the results in pre-monsoon summer daytime and nighttime were diametrically opposed. Daytime pre-monsoon summer has a negative SUHII due to less greenery in non-urban areas, which leads to greater land surface temperature and less evapotranspiration, potentially intensifying heat waves. When urban impacts are substantial, however, nocturnal pre-monsoon summer exhibited a favorable SUHII. Similarly, Alahmad et al. [31] investigated the spatial distribution of land surface temperature in Kuwait, comparing urban/suburban local government areas to rural and desert areas. During the day, the temperature difference between urban and rural areas was −1.1 °C, indicating that it is an urban cool island. However, the temperature difference rises to 3.6 °C at night, indicating the presence of an urban heat island, which the study goes on to suggest can be mitigated through urban planning policies. Zhou et al. [32] conducted a study in Europe and discovered that the larger the city, the greater the intensity of the UHI. However, the severity of the UHI decreases when the city has asymmetrical areas. Table 1 summarizes the current UHI and/or climate change status, which may deteriorate by 2050 if no mitigation techniques are implemented.

To prevent the UHI from worsening further, Singapore has implemented measures such as constructing green buildings and increasing green spaces. These green spaces include national parks, gardens and skyrise green rooftops, since greenery can help mitigate and/or slow down urban heat. Jusuf et al. [28] carried out qualitative and quantitative analysis to examine the impact of land use on Singapore’s temperature. Based on a qualitative study, they discovered that land with a significant concrete surface area, such as an airport, had a greater temperature during the day than at night. Quantitative research demonstrates that the industrial sector has a greater temperature than the Central Business District (CBD) area during the day and the opposite during the night. This is because the heat contained within the urban canyon progressively escapes into the environment. Green spaces, on the other hand, exhibited the lowest temperature during the day and a modest increase in temperature at night. Therefore, it is proven that the UHI can be reduced with proper land use planning. However, there is a paucity of research to determine whether strategies, such as constructing green buildings, skyrise greeneries, gardens, and national parks, can effectively decrease the UHI.

This paper focuses on the surface of the city (surface heat islands) and analyzes existing interventions in terms of greeneries and Green Mark commercial buildings on how it affects the temperature distribution based on non-exhaustive variables such as energy efficiency and consumption over a specific period. The research findings may be used to determine the type of greeneries that significantly contributes to the objectives of reducing building and surface temperatures. This study also highlights a significant correlation between the percentage change in temperature and concentration of Green Mark commercial buildings and green spaces across Singapore. Through this study, we also demonstrated the practical applications of using the ArcGIS software [34] for generating visual representations for analysis, which can also assist urban planners in formulating techniques, improve planning and inform policies to tackle the current situation.

## 2. Technical Review

We first provide a summary on the strategies and/or possible solutions to mitigate the UHI through various research papers, followed by the key factors that can affect temperature change. This includes discussion on Singapore’s mitigation strategies and showcasing details through ArcGIS software in later sections (Section 3.1).

### 2.1. Methodologies to Mitigate UHI

There are different types of strategies presented in numerous research papers to mitigate the UHI. China and Japan are focusing more on employing technologies to limit heat and carbon emissions, such as introducing electric vehicles and renewable energy [33]. City planners in Asia have been working hard to implement proactive methods to reduce the excessive heat stress caused by UHIs, which has been observed in several countries [1]. For instance, Edmundson et al. [35] conducted additional research on urban greenspaces based on soil surface temperature at 100 distinct locations. It was discovered that trees and shrubs could efficiently reduce soil temperature by 5.7 °C when compared to herbaceous plants in non-residential green areas. Zhang [36] investigated the cooling impact of the five most typically planted and prevalent varieties of shrubs in Guangzhou, China. The shrub, Murraya exotica, has the greatest temperature difference among the remaining four shrubs. This suggests that Murraya exotica may have the best cooling impact and may help to keep cities cooler. Wu et al. [37] presented a novel UHI mitigation technique based on the use of atmospheric aerosol particles (PM_2.5_) in Nanjing, which can alter the surface energy balance and the pace of atmospheric heating. When the environment is substantially polluted, a higher concentration of PM_2.5_ can lower UHI intensity by a maximum of 1 K. Due to particles’ radiative impacts, ambient temperature could also be effectively reduced by a maximum of 1.1 K. As a result, PM_2.5_ is useful in minimizing UHI and air pollution. Through the use of geographically weighted regression (GWR), remotely sensed photos, and street view images, Li et al. [38] investigated the effectiveness of the different types of plants, including grasses, shrubs, and trees, that are situated along the roadway depending on land surface temperatures. The research demonstrated that different parts of the Futian area have distinct cooling effects from grasses, bushes, and trees. Grass, shrubs, and trees, for example, are useful in commercial districts, industrial zones, and places highly inhabited with roads and buildings, respectively. As a result, urban greeneries have numerous environmental advantages in terms of improving air quality, reducing the UHI, and supporting a healthy urban ecology [39].

Other methods include green building construction to reduce carbon emissions and energy consumption [40], as buildings are a rising factor that increases anthropogenic heat, hastening the UHI process. In addition, utilizing green spaces in terms of proper landscaping [41,42,43,44], creation of urban green module design [45], and the application of cool pavement result in a significantly lower surface temperature and sensible heat flow to the atmosphere [46,47]. For instance, Jiang et al. [48] investigated the spatial morphology of Shanghai’s waterfront green space using mathematical statistics and spatial analysis techniques. Their spatial morphology findings reveal that the fractional cover values of the vegetation and size of the green space are significant parameters that may influence the cooling effect of the green space. Furthermore, their findings reveal that the broader the width of the river (more than 30 m), the colder the land surface temperature. Similarly, Shi et al. [49] conducted a seasonal SUHI analysis in Wuhan, China, and discovered that the compactness of buildings and trees had a different significant impact on land surface temperature. The land surface temperature will rise in closely built-up areas as opposed to densely populated areas with an abundance of trees. A similar study was carried out in Wuhan, where Xie and Li [50] discovered that due to the greater coverage of water bodies, nearly 90% of urban parks had the park cool island effect (PCI), with a PCI intensity ranging from 0.08 to 7.29 °C. Lu et al. [51] discovered that the UHI effect can be reduced by locating industrial sites away from residential regions and establishing buffer zones between commercial and residential areas. Li et al. [52] focused on using green and cool roofs to determine the effectiveness of reducing the UHI effect during the period of heat wave through the collaboration of Weather Research and Forecasting (WRF) and Princeton Urban Canopy (PUCM) models. Green roofs consist of soil and plants located on rooftops to increase the process of evapotranspiration and divert existing energy to latent heat. This results in cooling of the internal space, which reduces energy consumption [53,54]. On the other hand, cool roofs mainly help to improve roof surfaces with a higher albedo to reflect and prevent solar radiation from entering the building. These two types of roofs can minimize the amount of sensible heat being transmitted into the air and reduce the process of the UHI. Yang et al. [55] demonstrated a similar study through EnergyPlus simulations to prove that both types of roofs can reduce more than 30% of heat gains in Singapore. Based on these methodologies, we will be focusing on existing strategies used in Singapore to analyze how greeneries in terms of gardens, parks, and nature reserves as well as green buildings could help to mitigate and/or slow down the process of the UHI.

### 2.2. Greeneries

A study done by the National Parks Board (NParks) showed that Singapore has changed from carbon dioxide absorber in 2012 to carbon dioxide emitter in 2014. This is due to the combustion of fossil fuels, which contributes the most greenhouse gas emissions as an estimated 95% of electricity is produced from fossil fuels [56]. Thus, Nparks has been selectively planting species that can absorb more carbon dioxide to steadily reduce the emissions [57].

With a high density of both population and buildings, there is a need for additional innovative strategies to rejuvenate urban design [58]. For instance, green lush landscapes that are classified and maintained by NParks have more than 350 gardens, parks, and nature reserves in Singapore [59]. These spaces have a variety of heritage trees and biodiversity that significantly contribute to the control of climatic changes. Wong et al. [60] have studied the effectiveness of vertical greenery systems and discovered that it is helpful in lowering indoor temperature and cooling load energy consumption. With a 50% coverage of vertical greenery systems, it is possible to reduce the envelope thermal transfer value (ETTV) of a glass façade building by around 41%. As a result, the higher the coverage of vertical greenery systems, the lower the temperature and energy consumption of air-conditioning and mechanical ventilation (ACMV) systems.

In addition, Zhu et al. [61] states that urban parks are an effective method in mitigating the urban heat island effect. Their case study suggests that the larger the park size, the better the cooling effect due to the high concentration of plants. Yang et al. [62] conducted a similar study and agreed that urban parks could help to cool down the surrounding areas. For instance, the cooling effects could cover an estimated 480 m measured from the end of the park. This can happen when the area of the park is more than 30 ha. Wang et al. [63] investigated the cooling impacts of urban parks and realized that a combination of park landscape metrics such as park acreage and perimeter, greeneries, and waterbodies could significantly reduce land surface temperature. They discovered that increasing the distance between the park by a minimum of 100 m could significantly reduce the land surface temperature by an estimated 0.67 °C.

Due to land scarcity in Singapore, NParks has initiated an incentive scheme whereby existing buildings are landscaped with sustainable solutions from green facades, including rooftop gardens and vertical green walls that were started recently. By offsetting 50% of the developmental charges, the incentive till date has been utilized by more than 180 buildings and now the country boasts more than 133 hectares of greenery [64]. Furthermore, Zheng et al. [65] carried out simulations through EnergyPlus software to analyze whether green roofs could reduce the energy consumption and reduce the urban heat island effect in Los Angeles. Their results proved that green roofs could provide a cooling effect, which greatly reduce air-conditioning and mechanical ventilation (ACMV) energy consumption. This is due to the three key factors that aid in reducing building energy usage: amount of irrigation saturation, depth of soil, and Leaf Area Index (LAI). 

With these existing policies to rejuvenate urban design, such as having more than 350 gardens, parks, and nature reserves, Singapore aims to reduce 36% of greenhouse gas (GHG) emissions by 2030 to maintain its current emissions of 65 million tons and potentially reduce this amount by half in 2050 [66]. Wong et al. [67] indicates an urban temperature range of about 4 °C across Singapore due to the built infrastructures absorbing and trapping heat during the day and releasing it at night, which increases the temperature of its surroundings. In contrast, wetter vegetation and the shadowing effect of such plants may help to minimize heat through evapotranspiration [68]. Therefore, green spaces have a significant drop in heat emitted due to photosynthesis. Additionally, trees can block a significant amount of shortwave solar radiation, neutralize carbon dioxide and transform sensible heat into latent heat of vaporization [69]. Trees can also store heat in their trunks and roots to prevent carbon from releasing into the atmosphere again [56].

### 2.3. Green Buildings

An alternative strategy to mitigate and/or slow down the process of the UHI is to promote Green Mark commercial buildings as buildings consume an estimated 30% to 40% of total energy consumption [70,71,72,73,74]. To effectively cut the demand for electricity and use energy efficiently, the Buildings and Construction Authority (BCA) introduced a Green Mark certification scheme in 2005 to promote sustainable design in new and existing buildings [75]. This provides developers and building owners a scheme to improve and reduce the energy consumption while evaluating the environmental impact. For instance, Green Mark Commercial Buildings have effectively improved the energy utilization index (EUI) by an estimated 14% as compared to non-Green Mark buildings [76]. The Green Mark certification scheme [75] has been beneficial in better managing resources such as energy consumption and water use, and is deemed attractive in creating value for occupants in terms of health, property value, and branding. Buildings receive a rated award (Certified, Gold, GoldPlus and Platinum) based on an overall rating score from the six categories presented in Table 2.

With the Green Mark certification scheme, this encourages the building owners to have a minimum of 80% of their buildings Green Mark Certified by 2030 [75] as well as achieve green transformation through designing or retrofitting buildings with low reliance on traditional carbon-intensive cooling and air-conditioning.

In this paper, we provide an analysis on the impact of green spaces in terms of skyrise green rooftops, national parks, gardens, and Green Mark commercial buildings with reference to the temperature distribution in Singapore. The approach combines different types of datasets into maps to determine the efficiency of Green Mark commercial buildings and how these factors can help to mitigate heat and slow down the UHI in Singapore.

## 3. Materials and Methods

First, we obtained the relevant data during the pre-COVID period between the years 2017 to 2019. Second, the data were cleaned and imported into ArcGIS software for processing and for spatial analysis to create thematic maps. These thematic maps were provided for better visualization and interpretation on the current state of the UHI in Singapore. Furthermore, the thematic maps would aid in the investigation of the impact of green spaces and Green Mark commercial buildings on temperature distribution based on non-exhaustive variables in terms of energy efficiency and consumption, as well as how it mitigated UHI and influenced microclimate in Singapore. The method described is relevant and suitable for application by other forms of data science analysis and investigations.

### 3.1. Overview of Data Source and Methodologies

**Figure 1:**
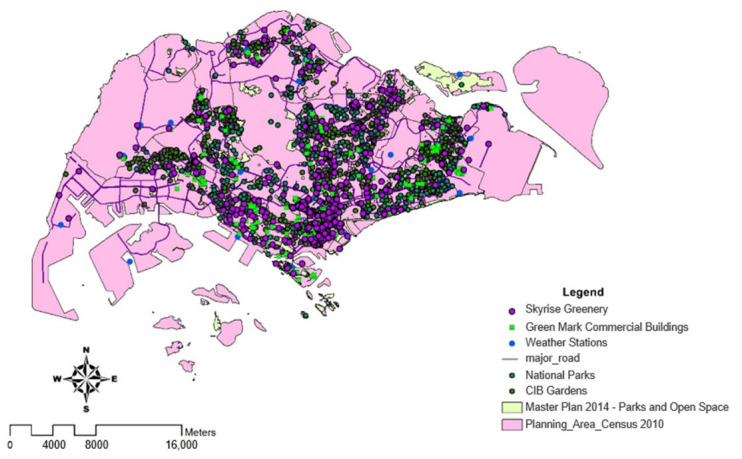
An overall illustration of Green Mark commercial buildings and facilities in Singapore. This map includes datasets of skyrise greeneries, Green Mark commercial buildings, weather stations, national parks (NParks) and Community in Bloom (CIB) gardens, parks and open spaces are displayed.

The data were obtained from Singapore’s Public Data to study the potential urban heat island effect area. For instance, the Building and Construction Authority (BCA) provided data on locations and energy consumption of Green Mark buildings based on voluntary disclosure. The National Environmental Agency (NEA) provided temperature data and location of national parks. The data comprised a Singapore map, including major roads, regional divisions based on census 2010, greeneries such as parks, skyrise greens, and nature reserves as well as locations of Green Mark buildings as shown in Figure 1. This map allowed us to analyze energy consumption and impact on the environment. With the voluntary details on Green Mark commercial buildings, we obtained the energy consumption and gross floor areas to compute energy consumption per square meter and compare all Green Mark buildings in all industry sectors such as retail, offices, and hotels in Singapore. Air temperature was obtained from various weather stations to analyze the effect of the UHI.

Urban forms of Singapore were created through geographic information system (GIS) mapping and geoprocessing tools. For this research, data were mapped with the physical attributes and building designs to digitally showcase the pattern of energy consumption and features of land-use spatial allocation [58]. Thematic maps were created for visual representations to provide insights on the possible critical factors that result in urban heat island effects such as dense building areas where there might be little or no evidence of greenspaces such as parks, gardens, and green rooftops in the area.

In addition, various charts were created through our subroutines written in Python, such as NumPy, Pandas, and Matplotlib. NumPy was imported to perform higher mathematical and statistical operations for multidimensional arrays and matrices. Pandas was imported to produce table-like data and allow us to carry out data cleaning. Matplotlib was imported to showcase various types of charts. With this information, we aim to propose workable solutions, such as revitalizing greenspaces in areas to provide further resilience towards climate change. To achieve this, we propose a simple and elegant method to be discussed in greater detail in the next section.

### 3.2. GIS Based Methodology

GIS data were first processed and imported into ArcGIS software for conversion into suitable objects. For instance, point objects were used as a label for Green Mark buildings, weather stations, and skyrise greenery rooftops, while polygon objects represent regions and park areas to showcase the temperatures in various locations across Singapore. Interpolation via geocoding could aid in the creation of a temperature layer by leveraging weather station data in spatial analysis, such as locations with a low percentage of temperature change. Next, to study the density of green spatial activities, the plotting of skyrise green, national parks, and Green Mark buildings, a vector spatial analysis tool was utilized to establish a buffering region with a 2 km radius around the weather station. A total of four buffer zone locations were evaluated by comparing the areas with the lowest percentage change to the areas with the highest percentage change, which is explained further in details in the next section. Cluster mapping generated a zoomed-in area to focus on the density of greeneries in Marina Barrage, Newton, Sembawang, and Tengah.

### 3.3. Data Processing

- Weather data

**Figure 2:**
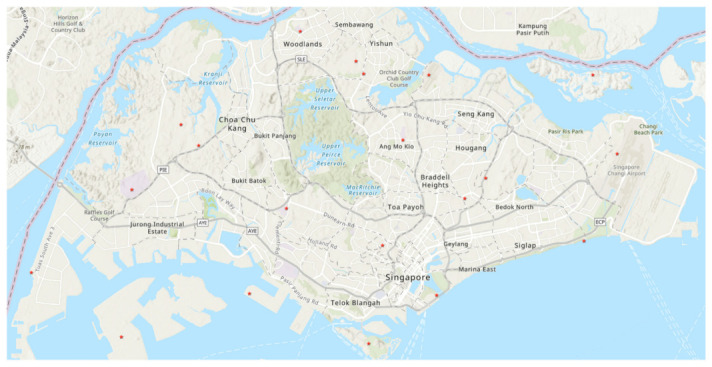
Based on longitude and latitude details, an overall illustration of the locations of the weather stations in Singapore was plotted in red stars [78].

Weather station point objects were generated with longitude and latitude details, as shown in Figure 2, which were obtained together with temperature data. The temperature difference between 2017 and 2019 and the percentage of temperature change were then calculated and showcased in thematic maps.

- Green Marked building data

A Green Marked building data excel sheet, obtained from Singapore’s Public Data, was imported into ArcGIS software to generate Green Marked Building point objects. It was then combined with an existing dataset that included all buildings in Singapore based on their postal code and duplicated data was removed. Geocoding was required to translate street addresses or other text information into coordinates that can be shown on a map. Interpolation was frequently used in geocoding to determine the location information for an address.

- Park data

The locations of national parks (NParks) were imported into ArcGIS software as an object file to include the geometry of all park areas. This helped to save much effort for the area-based calculations.

### 3.4. Spatial Analysis

Firstly, the spatial join method was used to find the number of Green Marked buildings, skyrise greeneries and the area of Community in Bloom (CIB) gardens and national parks that lie within a region. The regions were all based on the Planning Area Census 2010 obtained from Singapore’s Public Data.

**Figure 3:**
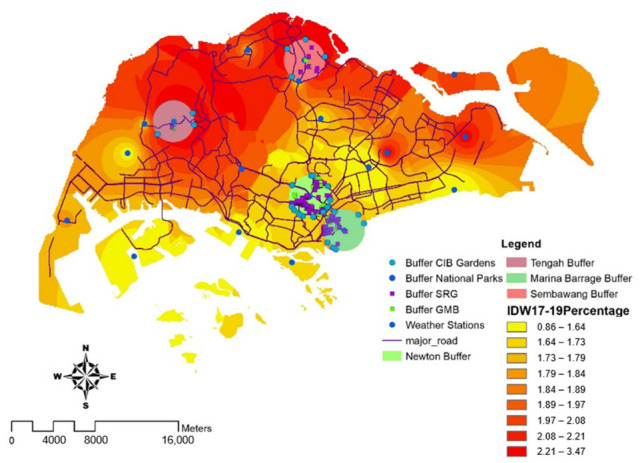
Interpolated map of the distribution of the percentage change in temperature with skyrise greenery (SRG), Green Mark buildings, national parks and gardens. Pink buffer zones are those with the greatest temperature change and the least amount of green area. Green buffer zones have the least temperature fluctuation and the highest proportion of green spaces.

Following that, buffering analysis was carried out, with four 2 km radius buffering regions measured from weather stations for comprehensive study, as shown in Figure 3. Two regions were chosen for having the lowest percentage of temperature rise while having the most GMB and greenery around the Marina Barrage and Newton weather stations. The other two regions were picked for having the largest percentage of temperature rise while having the fewest GMB and greeneries around the Sembawang and Tengah weather stations. The intersection tool was used to find all target objects that were within a 2 km range. These objects include Green Mark buildings, skyrise greeneries, Community in Bloom gardens and national parks (NParks). In summary, there were 1 Green Mark building, 1 skyrise greenery, 3 Community in Bloom gardens and 0 NParks in the Tengah area; 2 Green Mark buildings, 6 skyrise greeneries, 4 Community in Bloom gardens and 1 NParks in the Sembawang area; 16 Green Mark buildings, 32 skyrise greeneries, 7 Community in Bloom gardens and 0 NParks in the Marina Barrage area; 67 Green Mark buildings, 60 skyrise greeneries, 14 Community in Bloom gardens, and 0 NParks in the Newton area. Further details on the results are explained in Section 4.

Lastly, raster spatial analysis worked well for data collected from image sensors such as satellites. As all feature types were represented by the same cell-based structure, they could all be addressed the same way. This consistent structure enabled the combination of a wide range of geographic features in a single geoprocessing procedure. Therefore, raster spatial analysis was used for better illustration of the temperature distribution in Singapore since there were a total of eighteen weather stations for temperature data. Due to the lack of data points in some weather stations, we chose to interpolate the temperature between the two years. For example, we would subtract the greatest and lowest temperatures and divide the result by two. Depending on the year, we then added or subtracted the temperature differential. For instance, we added the difference in temperature from the year 2017 if the missing temperature was in 2018. This was good enough for the interpolation because the weather stations were evenly distributed and the average temperatures have a small variance. Next, a raster image is a collection of grid cells, like a scanned map or picture, that is useful for showing continuous change such as temperature change. The cell size was set at 100 m to provide higher resolution for the dimension of the region covered on the ground and represented by a single pixel.

## 4. Results

We first present an overview of the results, followed by specifics on the locations of green spaces and Green Mark commercial buildings, beginning with an analysis on national parks (NParks), gardens, skyrise green rooftops, Green Mark buildings and lastly, a map of Singapore’s temperature data in 2019. The percentage change in temperature was calculated using data from weather stations in the year of 2017, 2018, and 2019. The temperature changes are correlated with three categories of urban spaces individually. These include national parks and gardens, skyrise green rooftops, and Green Mark commercial buildings.

### 4.1. A Summary of the Results

A combination of data on the impact of all greenspaces (skyrise greenery, national parks and gardens and Green Mark buildings) was analyzed with respect to the percentage change in temperature distribution from 2017 to 2019, based on the results as shown in Figure 3. In this map, we used inverse distance weighted (IDW) interpolation that calculates cell values by employing a linearly weighted combination on percentage of temperature change in Singapore. The heatmap includes all weather station locations, skyrise greeneries, and major roads, along with Green Mark buildings.

**Figure 4:**
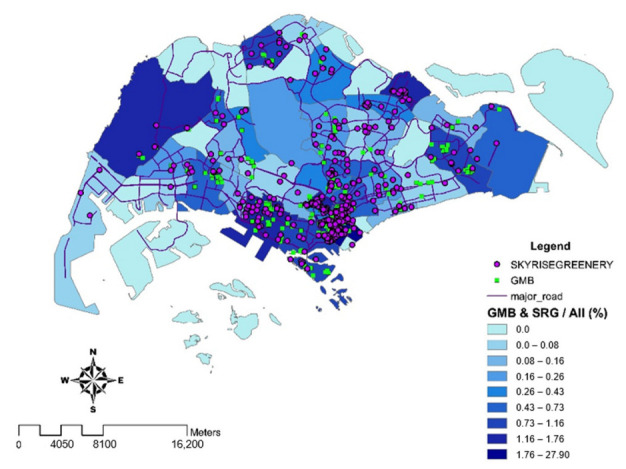
Spatial distribution of the percentage on a combination of both skyrise greenery (SRG) and Green Mark commercial buildings (GMB). Commercial districts such as Marina Barrage have an estimated 27.9% of GMB and SRG. This classification is based on Jenks’ Natural Breaks algorithm. Class breakdowns are determined for best group comparable data and maximize the differences across classes of SRG and GMB.

A 2 km buffer zone was generated around the highest and the lowest concentration of green spaces and Green Mark commercial buildings. It was discovered that the local climatic zone (LCZ) in northern Singapore (pink buffer zones), such as Tengah and Sembawang, was considered open high-rise, with a low number of Green Mark commercial buildings and a low concentration of green spaces. Thus, the percentage of temperature change was the highest with an estimated 2.2%. In contrast, the LCZ for the central business district, such as Marina Barrage and Newton, where there is a dense mix of Green Mark commercial buildings, was considered as compact high-rise. However, the LCZ for the central business district was also regarded as dense trees, with the largest proportion of green spaces. Therefore, Marina Barrage and Newton were recorded with the least percentage of temperature change, an estimated 1.6% (green buffer zones). Based on the results of Figure 3, it is essential to have a combination of high concentration of greeneries such as skyrise greenery, national parks, gardens and Green Mark buildings especially in CBD areas such as Marina Barrage and Newton, as illustrated in Figure 4, which has the least percentage change as compared to non-central areas, to further mitigate the UHI effect.

Cluster maps were generated to see the concentration of factors in a buffered zone and analyze the relation between the density of green spaces (buildings, skyrise greenery and gardens) and the impact it has on temperature change. The concentration ratio of Green Mark buildings and other green spaces in non-central areas that have experienced the highest percentage change in temperature will be explored in the later part of this paper.

### 4.2. National Parks and Gardens

**Figure 5:**
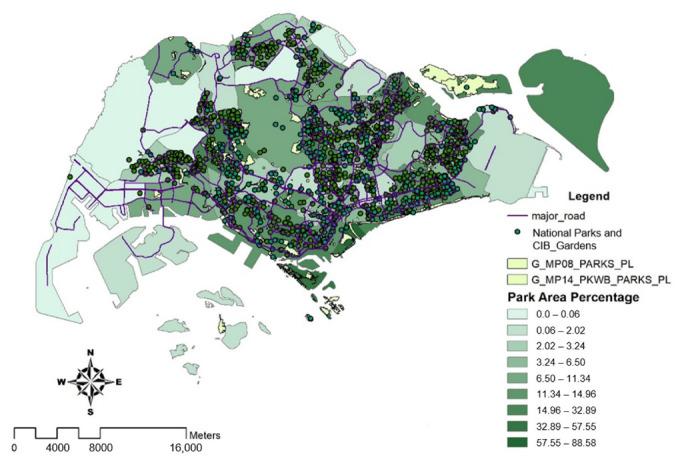
Spatial distribution of park area percentage. The map displays the total percentage of parks and CIB gardens across the region using the data-specific classification technique ‘Natural Breaks’, with dark colors representing a high percentage of park area and light colors representing either none or a small percentage of park area.

Figure 5 displays the concentration of green parks and Community in Bloom (CIB) gardens around Singapore. This visualization further supports the argument that areas where temperatures were recorded to have the highest percentage change, particularly in the northern regions such as Tengah, Sembawang, and Khatib, have a smaller percentage of green park spaces and gardens (with a maximum of about 11%). With approximately more than 57.5% of green areas in commercial districts such as Marina Barrage and Newton, they have the least percentage change in temperatures. In addition, East Coast Parkway and central eastern regions also have a higher density of parks and greeneries.

### 4.3. Skyrise Green Rooftops

**Figure 6:**
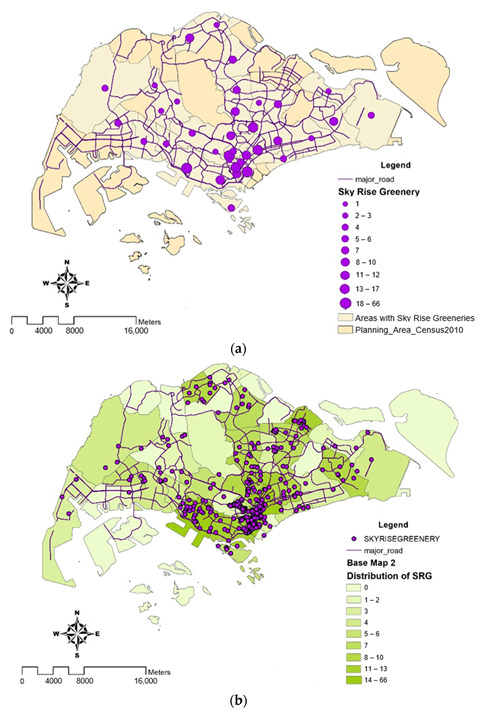
(**a**) The map displays the cluster of skyrise greenery (SRG) rooftops where larger cluster circle represents the higher number of green rooftops in a planning area. (**b**) Concentration of skyrise greenery (SRG) rooftops. The map displays the distribution of skyrise greenery rooftops using the classification technique ‘Natural Breaks’, with dark colors indicating large concentration of skyrise greenery rooftops and light colors indicating either no or tiny concentration of skyrise greenery rooftops.

Figure 6a,b illustrates the number of skyrise green rooftops registered by NParks, as presented in cluster and distribution maps. It is evident from the maps that the greatest number of clusters can be observed in the commercial districts, Marina Barrage and Newton. Areas with least clusters observed were situated in the northern regions and other non-central areas.

### 4.4. Density of Green Mark Building Clusters

**Figure 7:**
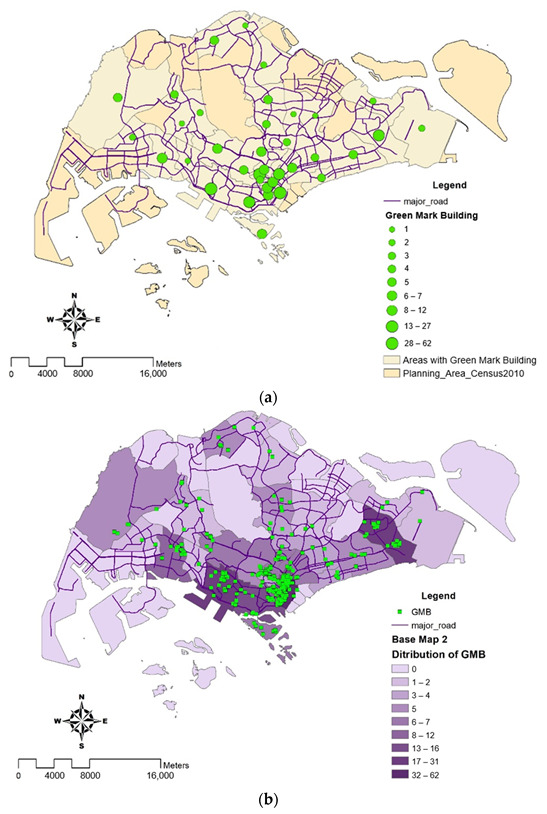
(**a**) The map displays the cluster of Green Mark Buildings (GMB) where larger cluster circle represents the higher number of GMB in a planning area. (**b**) Concentration of Green Mark Buildings (GMB). The map displays the distribution of GMB using the classification technique ‘Natural Breaks’, with dark colors indicating large concentration of GMB and light colors indicating either no or tiny concentration of GMB.

Figure 7a,b illustrates the cluster and concentration of Green Mark certified buildings by planning area. The information provided from both maps predominantly show a higher density of Green Mark buildings in Marina Barrage and Newton. As major roads were illustrated in purple lines, it could be seen that there is a marginal increase in Green Mark buildings, which could help to reduce the urban heat island effect. However, this may not be conclusive as there are not enough weather stations situated along major roads to identify the temperature difference.

### 4.5. Temperature

#### 4.5.1. Annual Temperature in 2019

**Figure 8:**
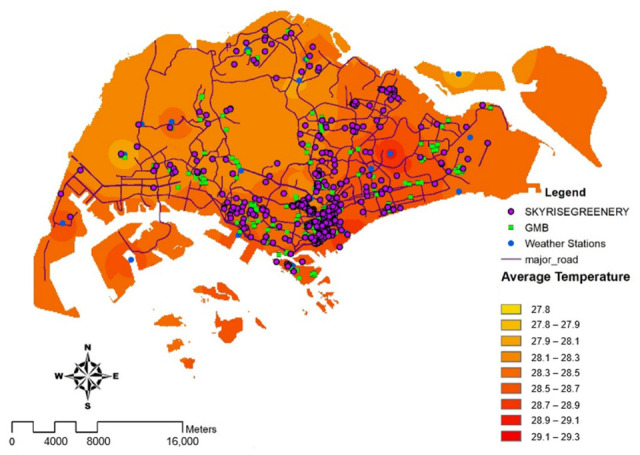
Annual Average Temperature (℃) in the year of 2019 in Singapore. Interpolation is used to calculate the average temperature based on a set of data obtained from meteorological stations.

Table 3 and a choropleth map, as displayed in Figure 8, reveal that the greatest temperatures recorded in 2019 were largely situated in commercial business sectors such as Marina Barrage, Newton, Changi, and the central northern region, Paya Lebar. A potential reason is due to a high density of commercial activities (offices and retail). Low temperatures were observed in islands such as Pulau Ubin and Jurong West due to the high number of greeneries such as national parks.

#### 4.5.2. Annual Percentage Temperature from 2017 to 2019 in Singapore

**Figure 9:**
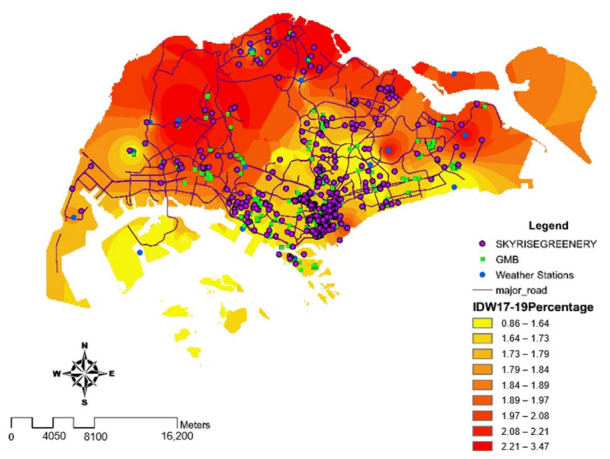
Temperature heatmap to show increase in percentage change in Singapore from 2017 to 2019. Inverse distance weighted (IDW) interpolation is used to calculate the percentage change in temperature based on a set of data calculated from meteorological stations using a linearly weighted combination.

Figure 9 illustrates a heatmap with the highest percentage increase in temperature across the region. The northern regions of Singapore experienced the greatest percentage change in temperature (an approximation of 3.47%), such as Khatib (2.7%), Sembawang (3.2%), and Tengah (3.5%), whereas the southern-central regions experienced the smallest percentage change in temperature (an approximation of 1.84%). This is possibly due to the high percentage (an estimated 27.9%) of skyrise greeneries and Green Mark certified buildings as shown in Figure 4.

Although the average temperatures between 2017 to 2019 were consistently recorded higher for areas that have high commercial activity, such as Marina Barrage, Newton, Paya Lebar and Changi, the increase in percentage change is significantly lower at an estimated 2.5%, where Marina Barrage recorded the lowest among the three commercial districts. In addition, non-commercial districts have recorded low percentage changes in temperature, such as East Coast Parkway, Pulau Ubin, and Tai Seng. This can be potentially due to the high concentration of green spaces in these areas.

## 5. Discussion

Due to a high number of green spaces and Green Mark commercial buildings, our research indicates that commercial districts have the lowest percentage of temperature change when compared to non-commercial districts, with an estimated 1.6%. According to this study, there is a substantial link between the amount of green space present across Singapore and the percentage change in temperature. Particularly in non-commercial areas, there is a need to create a range of green areas, such as gardens and skyrise greeneries, which may help to lessen urban heat. Our study contributes to bridging the knowledge gaps in evaluating the effectiveness of Green Mark commercial buildings, skyscraper greeneries, gardens, and national parks.

Our findings indicate that having a high density of Green Mark buildings in the CBD region does not worsen the UHI effect, which contradicts a major aspect of having densely inhabited buildings in an urban area that contributes to UHI. The densely populated buildings limit the wind speed and increase thermal capacity. Instead, it is effective in slowing down and/or maintaining the temperature change. This is because Green Mark buildings are designed with energy-efficient lighting and ACMV, and built with recycled materials that do not affect thermal comfort and reduce energy consumption. For instance, more than 60% of Green Mark buildings have been awarded a minimum of GoldPlus among a total of 115 Green Mark buildings located in Marina Barrage, as illustrated in Table S1. The GoldPlus and Platinum awards assisted the owners in either maintaining or reducing energy consumption per square meter based on the comparison from 2017 to 2019. Only a small percentage of the buildings had minor increases in energy consumption per square meter, which may have been brought on by a change in operating hours or a decline in the energy efficiency of equipment such as ACMV after a few years of use. Therefore, the Green Mark certifications are only valid for five years, allowing building owners to replace their machinery while also increasing their buildings’ energy efficiency. On the other hand, non-central areas such as Khatib have three Green Mark commercial buildings (2 Platinum and 1 Gold awards). There is a slight increase in energy use intensity as illustrated in Table 4. This could be due to the low percentage of parks (an estimated maximum of 6.5%) and the high percentage of temperature change (an estimated 2%) as shown in Figure 9, resulting in higher energy consumption to maintain the thermal comfort for the building occupants. Therefore, with a higher Green Mark award, energy consumption can be reduced and having a high concentration of Green Mark buildings in an area is useful in reducing and/or maintaining the temperature change.

**Figure 10:**
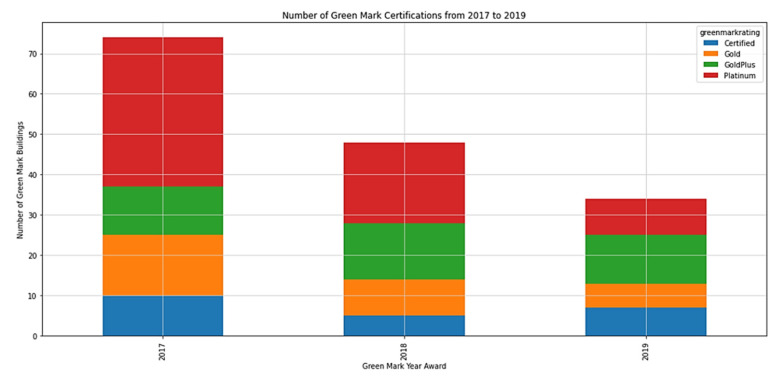
Number of registered Green Mark buildings from 2017 to 2019.

Aside from focusing on Green Mark commercial buildings located in Marina Barrage and Khatib, there was a spike in Green Mark buildings registered in 2017, as illustrated in Figure 10, due to an amendment in regulations that requires all buildings (except those industry sectors in railings, industrial, data centers, religious, utility and residential buildings) with a centralized cooling system and gross floor area more than 5000 m^2^ to install and/or replace ACMV in their buildings [80].

In addition, the buildings that applied before 1 April 2019 were given a three-year grace period to fulfil the criteria stated in the new Green Mark existing non-residential building 2017 version (GM ENRB: 2017) [81]. Thus, there might be an increase in the number of Green Mark buildings from 2020 onwards.

**Figure 11:**
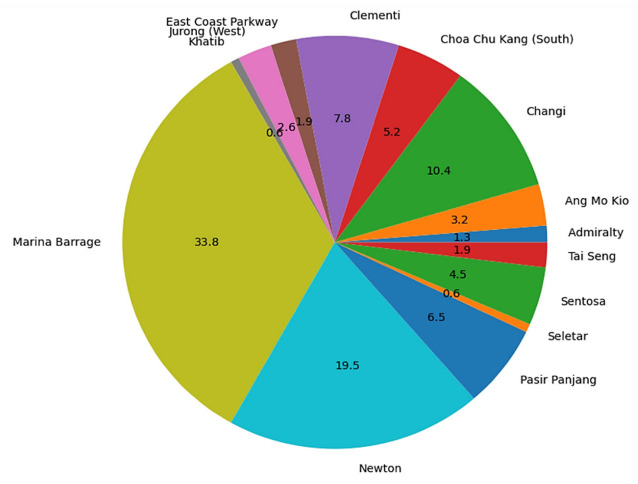
Cumulative distribution of Green Mark Buildings registered in Singapore (rounded up to 1 decimal point).

With reference to Figure 11, the highest number of Green Mark buildings are in Marina Barrage (33.8%), Newton (19.5%), and Changi (10.4%) weather stations. It is seen that the number of Green Mark buildings registered are relatively lower in non-commercial areas as compared to commercial areas. This also means that there is a high density of Green Mark buildings in the central areas, as seen in Figure 7.

**Figure 12:**
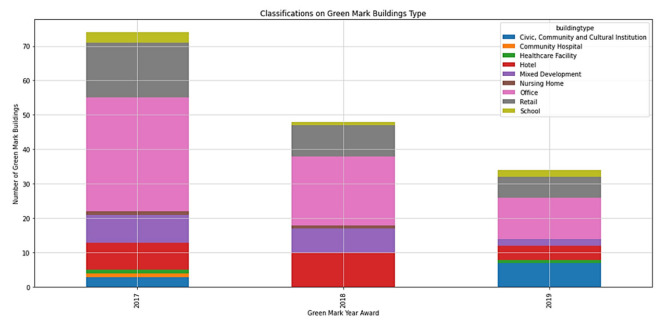
Classifications of Green Mark buildings in various industry sectors.

**Figure 13:**
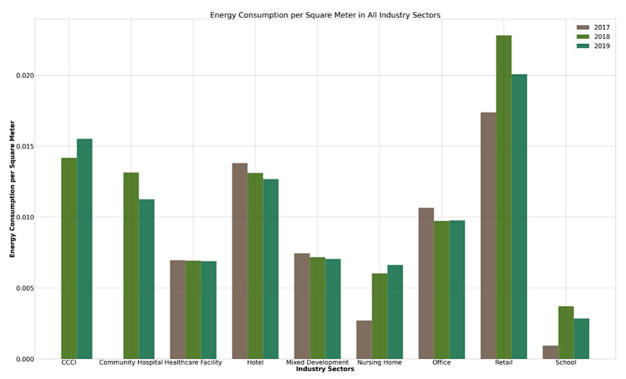
Energy consumption of commercial industry sectors per square meter.

With reference to Figure 12, offices have the highest number of Green Mark registrations among all industry sectors, followed by retail and hotel sectors, which also correlates to having the highest energy consumption per square meter as seen in Figure 13 below. However, retail has the highest energy consumption reduction per square meter, followed by the hotel and office sectors. This could be potentially due to different operating hours in each industry sector. Most importantly, the retail sector has the lowest percentage of Platinum and GoldPlus awards as compared to the office and hotel sectors, as seen in Table 5.

Next, according to NParks, urban skyrise greeneries are expanding in order to alleviate urban heat [82]. The incentive schemes announced by Nparks have encouraged the implementation of greenery, hence the evident number of clusters in the commercial districts (Marina Barrage and Newton). The types of skyrise greenery could not be categorically presented, but it would be interesting to see which types of greenery significantly contributes to the objectives of reducing building and surface temperatures as part of future work.

In addition, our analysis could help urban planners formulate techniques to mitigate and/or slow down the urban heat island effect and further enhance sustainability to eliminate the bottleneck of expensive construction costs and environmental damage in urban design and construction. For instance, Xiao et al. [83] investigated the spatial relationship between the microclimate and green spaces in a total of 15 parks located in Suzhou Industrial Park (SIP) and realized that medium-sized green spaces can provide some cooling and humidifying impact during hot days. Quaranta et al. [84] presented various benefits of urban greening, such as reducing 55.8 Mtons/year of CO_2_, 92 TWh/year of ACMV energy consumption, and an estimated 2.5 to 6 °C in Europe. Not limited to only the external environment, urban greening also brings great benefits to biodiversity, quality of water, and humans in terms of health and wellbeing.

There are some limitations in the methodological approach that could be addressed in future work. Due to confidentiality, there are limited or no details on the updates of regulations and external facades; thus, both are not considered to be part of this paper. One of the limitations concerns the plotted year, as the Green Mark commercial buildings data, retrieved from the Building and Construction Authority (BCA), are only available from 2017 to 2019. The data also consist of a combination of voluntary and non-voluntary disclosure on the details of Green Mark buildings such as locations and energy consumption. As no details are provided for non-voluntary disclosure, we have therefore filtered and used voluntarily registered information for this study. Although this may not be an entire representation, it is sufficient to be used as sample data to ascertain the relationship with temperature and heat effects.

Next, the temperature data of Green Mark buildings are obtained from the nearest weather stations. However, the weather station located in Seletar does not record any temperature data; therefore, we were unable to include it in the map. To overcome this limitation, we estimated the temperature to complete the dataset since there are some Green Mark buildings located near the Seletar weather station.

As the Green Mark certifications are only valid for five years and the energy consumption of buildings may increase due to the deterioration of equipment’s energy efficiency over a certain timeframe, there are limited data for monitoring its validity for assessing long-term benefits of mitigating urban heat. Therefore, we could look at other alternatives for mitigating urban heat, such as harvesting energy from solar irradiance to bring clean electricity to buildings while reducing the solar energy absorbed by the building and surrounding air.

## 6. Conclusions

We divided the data analysis into five parts, starting with a summary of the findings, then moving on to national parks (NParks), gardens, skyrise green rooftops, Green Mark buildings, and finally, a map of Singapore’s temperature data in 2019. We analyzed the green spaces and energy consumption to effectively discuss the buffer zone interpolation heatmap as shown in Figure 3. This study highlights a significant correlation between the percentage change in temperature and concentration of green spaces across Singapore. There is a need to increase a variety of green spaces such as gardens and skyrise greeneries, especially in non-commercial districts, which may aid in the reduction of urban heat. This is because greeneries have greatly contributed to the goals of lowering building and surface temperatures, as validated by Wong et al. [60] and Zhang [36]. Further research can be carried out in focusing on the types of greeneries found in commercial districts that are effective in lowering the percentage of temperature change. Next, a highlight of our findings suggests that higher density and diversity of Green Mark buildings, such as in Marina Barrage (an estimated 27.9%), can aid in reducing temperature change, regardless of the types of commercial buildings, which may have varying energy consumption. As a result, instead of focusing solely on greeneries, our research findings suggest that combining Green Mark buildings and green spaces can assist in reducing UHI and environmental degradation while also preventing catastrophic effects in 2050, as shown in Table 1. Our study also contributes to filling the research gaps in identifying the effectiveness of Green Mark commercial buildings, skyrise greeneries, gardens, and national parks. This influences microclimate change and mitigates UHI in a tropical city like Singapore. We also believe that our proposed methodology can be extended to other relevant countries.

## Figures and Tables

**Table 1:** A summary of the current situation in various parts of Asia, as well as the ramifications if nothing is done to prevent UHIs and/or climate change by 2050 [33].

Which Part of Asia?	Examples of Countries	Current UHI and/or Climate Change Situation	Consequences If No Action Is Taken by 2050 to Reduce UHIs and/or Climate Change
Frontier Asia	Bangladesh Pakistan India	Severe heat and humidity can have a substantial impact on their comfort and ability to work.	The average temperature is expected to rise by a minimum of 2 °C and a maximum of 4 °C. Increased likelihood of fatal heat waves. Extreme precipitation occurrences are becoming increasingly common.
Emerging Asia	Malaysia Thailand Singapore Cambodia Vietnam Indonesia	Most of the countries will suffer severe heat and humidity.	Severe heat and humidity Increased likelihood of precipitation occurrences.
Developed Asia	South Korea Australia New Zealand Japan	Climate change is expected to be less likely in Developed Asia as compared to Frontier and Emerging Asia. Some countries will suffer water availability and drought challenges.	Drought conditions in southern Australia are expected to worsen by more than 80%. Beginning in 2030, wildfires will become increasingly common especially in southern Australia.

**Table 2:** Green Mark certification rating categories [75,77].

**Green Mark Certification Rating Categories**	Energy Efficiency
	Water Management
	Material and Waste Management
	Environmental Planning
	Green Buildings and Transport Infrastructure
	Community and Innovation

**Table 3:** Comparison of 2019 average temperature data across Singapore [79].

Locations of Weather Stations	Average Temperature (°C)
Changi	28.4
Paya Lebar	29.1
Tengah	28.6
Seletar	28.8
Sembawang	28.4
Tai Seng	28.5
Jurong (west)	27.8
Clementi	28.1
Sentosa Island	28.6
Admiralty	28.1
Pulau Ubin	27.8
East Coast Parkway	28.4
Marina Barrage	29.0
Ang Mo Kio	28.2
Newton	28.0
Tuas South	28.6
Pasir Panjang	28.6
Jurong Island	28.7
Choa Chu Kang (South)	28.1
Khatib	27.9

**Table 4:** Comparison of Green Mark buildings’ energy use intensity located in Khatib in the years of 2017, 2018, and 2019.

Building Name	Green Mark Award	Energy Use Intensity		
		2017	2018	2019
Khoo Teck Puat Hospital	Platinum	311	311	318.8
Yishun Community Hospital	Platinum	203	209	-
Northpoint City	Gold	468	594	587.7

**Table 5:** Comparison of Platinum and GoldPlus awards in hotel, retail, and office sectors.

Green Mark Award	Hotel	Retail	Office
GoldPlus	31.8%	22.6%	27.7%
Platinum	36%	35%	43.1%

## Data Availability

The data presented in this study can be found at data.gov.sg (accessed on 22 March 2021). The Python codes for Figure 10, Figure 11, Figure 12 and Figure 13 are uploaded to Open Science Framework, which can be found in https://osf.io/bktvu/?view_only=831a4c4847494bcba42d901194c59990 (accessed on 30 August 2022).

## Supplementary Materials

The following supporting information can be downloaded at: https://www.mdpi.com/article/10.3390/ijerph191911917/s1, Table S1: Comparison of Green Mark Buildings’ Energy Consumption per Square Meter in Marina Barrage.
