# Supplementary material for: Urban Heat Island Mitigation: GIS-Based Analysis for a Tropical City Singapore
Source: Int J Environ Res Public Health. 2022 Sep 21;19(19):11917. doi: 10.3390/ijerph191911917 (PMC9565339; doi:10.3390/ijerph191911917)
Supplement: Supplementary file 1 [file ijerph-19-11917-s001.zip › ijerph-1862808-supplementary.pdf]

Table S1. Comparison of Green Mark Buildings' Energy Consumption per Square Meter in Marina Barrage

**Building Name**	**Building Address**	**Building Type**	**Green Mark Rating**	**2017 Energy Consumption per Area**	**2018 Energy Consumption per Area**	**2019 Energy Consumption per Area**
Marina Bay Sands	1 Bayfront Avenue, Singapore 018971	Mixed Development	Platinum	0.000518	0.000510738	0.000519154
Suntec City Mall	3 Temasek Boulevard, Singapore 038983	Office	Gold	0.000656	0.000667277	-
Asia Square Tower 1	8 Marina View, Singapore 018960	Office	Platinum	0.00117	0.001194645	0.00114972
One Raffles Quay	1 Raffles Quay, Singapore 048583	Office	Platinum	0.0019	0.001734798	0.002015448
Tanjong Pagar Centre	7 Wallich Street, Singapore 078884	Office	Platinum	0.00154	0.001904013	0.001944157
Bugis Junction	230 Victoria Street, Singapore 188024	Mixed Development	Platinum	0.00321	0.003176898	0.003128705
One Raffles Place	1 Raffles Place, Singapore 048616	Office	Gold	0.00202	0.001752565	0.001734007
Oue Downtown	6 Shenton Way, Singapore 068809	Office	Certified	0.00137	0.001559605	0.001577516
South Beach Tower	26 Beach Road, Singapore 189768	Mixed Development	Platinum	0.00236	0.002363325	0.002372693
International Plaza	10 Anson Road, Singapore 079903	Office	GoldPlus	0.0016	0.001512649	0.001526709
Police Cantonment Complex	391 New Bridge Road, Singapore 088762	Commercial Building	Platinum	-	0.002218024	0.002110923
Esplanade Mall	8 Raffles Avenue, Singapore 039802	Retail	Platinum	0.0029	0.00247692	-
Republic Plaza	9 Raffles Place, Singapore 048619	Office	Platinum	0.00207	0.001787878	0.00198141

Singapore Management University	50 Stamford Road, Singapore 178899	University	Platinum	0.00143	0.00144454	-
Uob Plaza	80 Raffles Place, Singapore 048624	Office	GoldPlus	0.00275	0.00289638	0.002697749
The Metropolis	9 North Buona Vista Drive, Singapore 138588	Office	Platinum	0.00211	0.002068015	0.002117757
Asia Square Tower 2	12 Marina View, Singapore 018961	Office	Platinum	0.00208	0.002088857	0.002067323
Ocean Financial Centre	10 Collyer Quay, Singapore 049315	Office	Platinum	0.00217	0.002093924	0.002191441
Axa Tower	8 Shenton Way, Singapore 068811	Office	Platinum	0.00163	0.001579085	0.00165225
Capitagreen	138 Market Street, Singapore 048946	Commercial Building	Platinum	-	0.002147325	0.002188978
OCBC Centre	65 Chulia Street, Singapore 049513	Office	Gold	0.00266	0.002650541	0.002663098
The Plaza	7500a Beach Road, Singapore 199591	Mixed Development	Gold	0.00335	0.003387041	0.003517508
Duo Tower	3 Fraser Street, Singapore 189352	Office	Platinum	0.00187	0.003750959	0.004160368
National Gallery Singapore	1 St. Andrews Road, Singapore 178957	Civic, Community and Cultural Institution	GoldPlus	-	0.002742082	0.002675605
The Concourse	300 Beach Road, Singapore 199555	Office	Platinum	0.00215	0.002115656	0.002148205
National Library Building	100 Victoria Street, Singapore 188064	Office	Platinum	0.00376	0.003844649	-
Mas Building	10 Shenton Way, Singapore 079117	Office	Platinum	0.00252	0.002354817	0.002349942

The Ura Centre	45 Maxwell Road, Singapore 069118	Office	Platinum	0.00281	0.002700953	0.002905644
Concorde Shopping Centre	317 Outram Road, Singapore 169075	Hotel	GoldPlus	0.00494	0.003996891	0.003934592
Grand Copthorne Waterfront	392 Havelock Road, Singapore 169663	Hotel	Platinum	0.00505	0.004949155	0.004969246
One George Street	1 George Street, Singapore 049145	Commercial Building	GoldPlus	-	0.004143365	0.004161648
Chinatown Point	133 New Bridge Road, Singapore 059413	Mixed Development	Certified	0.00668	0.00623898	0.006903615
Peninsula Shopping Complex	3 Coleman Street, Singapore 179804	Commercial Building	GoldPlus	-	0.00471615	0.004464864
Jurong Point	1 Jurong West Central 2, Singapore 648886	Retail	Gold	0.0102	0.01002055	-
Fuji Xerox Towers	80 Anson Road, Singapore 079907	Office	Platinum	0.00347	0.003327309	0.003224721
Raffles Hospital	585 North Bridge Road, Singapore 188770	Healthcare Facility	Certified	-	0.009876308	0.006005296
People's Park Complex	1 Park Road, Singapore 059108	Commercial Building	Gold	-	0.005941832	0.005667158
Great Eastern Centre	1 Pickering Street, Singapore 048659	Office	Certified	0.0046	0.004510998	0.003557026
High Street Centre	1 North Bridge Road, Singapore 179094	Office	Platinum	0.00405	0.003837209	0.002591497
The Treasury	100 High Street, Singapore 179434	Office	Platinum	0.00342	0.003228993	0.003154156
The Adelphi	1 Coleman Street, Singapore 179803	Mixed Development	Platinum	0.00559	0.003887458	0.003697742
Oue Bayfront	50 Collyer Quay, Singapore 049321	Office	Gold	0.00737	0.007144623	0.007122716

Income At Raffles	16 Collyer Quay, Singapore 049318	Commercial Building	GoldPlus	-	0.006300786	0.00585531
Swissotel Merchant Court Singapore	20 Merchant Road, Singapore 058281	Hotel	Platinum	0.00547	0.005518474	0.005391141
Furama Riverfront Singapore	405 Havelock Road, Singapore 169633	Hotel	Platinum	0.00607	0.006813947	0.006750674
Golden Mile Tower	6001 Beach Road, Singapore 199589	Commercial Building	Gold	-	0.004140408	0.004190982
One Raffles Link	1 Raffles Link, Singapore 039393	Office	GoldPlus	0.0114	0.010639471	0.010373243
Capital Square	23 Church Street, Singapore 049481	Commercial Building	GoldPlus	-	0.009069843	0.009621047
Mapletree Anson	60 Anson Road, Singapore 079914	Office	Platinum	0.00603	0.005889447	0.005485825
Health Promotion Board	3 Second Hospital Avenue, Singapore 168937	Office	Platinum	0.00526	0.005101406	-
Ministry Of Manpower Building	18 Havelock Road, Singapore 059764	Office	Platinum	0.00418	0.004307923	0.004291511
Parkroyal On Pickering	3 Upper Pickering Street, Singapore 058289	Hotel	Platinum	0.0103	0.010572416	0.0109787
Novotel Singapore Clarke Quay	177a River Valley Road, Singapore 179031	Hotel	GoldPlus	0.00858	0.008406894	0.008476276
Prudential Tower	30 Cecil Street, Singapore 049712	Office	GoldPlus	0.00632	0.004946027	0.0057477
Bugis+	201 Victoria Street, Singapore 188067	Commercial Building	Platinum	-	0.0176813	0.01699945
Samsung Hub	3 Church Street, Singapore 049483	Office	GoldPlus	0.00646	0.006567013	-
79 Anson Road	79 Anson Road, Singapore 079906	Commercial Building	GoldPlus	-	0.005019504	0.005778224

Old Hill Street Police Station	140 Hill Street, Singapore 179369	Office	Platinum	0.008	0.005126832	0.005496883
Furama City Centre Singapore	60 Eu Tong Sen Street, Singapore 059804	Hotel	Platinum	0.00909	0.008972425	0.009035875
OG Albert Complex	60 Albert Street, Singapore 189969	Mixed Development	Gold	0.00731	0.007036231	0.006694283
Fortune Centre	190 Middle Road, Singapore 188979	Office	Gold	0.00755	0.007593621	-
Hotel Miramar	401 Havelock Road, Singapore 169631	Commercial Building	Gold	-	0.007822939	0.007841245
Twenty Anson	20 Anson Road, Singapore 079912	Commercial Building	Platinum	-	0.008573892	0.008939064
Tong Eng Building	101 Cecil Street, Singapore 069533	Office	Platinum	0.00541	0.004928042	0.004967567
Sim Lim Tower	10 Jalan Besar, Singapore 208787	Mixed Development	GoldPlus	0.00593	0.005620938	0.005663436
OCBC Center East	63 Chulia Street, Singapore 049514	Office	Gold	0.0087	0.008926192	0.008277529
SMU Administration Building	81 Victoria Street, Singapore 188065	University	Platinum	0.00618	0.006044356	-
Pomo	1 Selegie Road, Singapore 188306	Private School	Platinum	0.0132	0.011954929	-
Four Points By Sheraton Singapore, Riverview	382 Havelock Road, Singapore 169629	Commercial Building	Gold	-	0.013989667	0.013757145
Nanyang Academy Of Fine Arts (NAFA) Campus 1	80 Bencoolen Street, Singapore 189655	Private College	Gold	0.011	0.012709444	0.011748739
Grand Park City Hall	10 Coleman Street, Singapore 179809	Commercial Building	Platinum	-	0.008784952	0.009322167
Carlton City Singapore	1 Gopeng Street, Singapore 078862	Commercial Building	Platinum	-	0.013402582	0.013303532

Parliament House Complex	1 Parliament Place, Singapore 178880	Office	Platinum	0.00643	0.006678472	0.006493633
Rendezvous Hotel Singapore	9 Bras Basah Road, Singapore 189559	Hotel	GoldPlus	0.0148	0.015618661	0.014794186
The Riverwalk	20 Upper Circular Road, Singapore 058416	Commercial Building	Certified	-	0.013539983	0.012887181
MYP Centre	9 Battery Road, Singapore 049910	Office	Gold	0.0108	0.010449771	0.009205712
Copthorne King's Hotel	403 Havelock Road, Singapore 169632	Hotel	Platinum	0.0136	0.01420616	0.014236858
Health Sciences Authority Building	11 Outram Road, Singapore 169078	Commercial Building	GoldPlus	-	0.025937182	0.0262939
Health Sciences Authority Building	11 Outram Road, Singapore 169078	Office	GoldPlus	0.0259	0.025919926	-
Maxwell House	20 Maxwell Road, Singapore 069113	Office	Platinum	0.00921	0.007466281	-
The Octagon	105 Cecil Street, Singapore 069534	Office	Certified	0.00783	0.006965849	-
Keck Seng Tower	133 Cecil Street, Singapore 069535	Office	Certified	0.00912	0.008559732	0.008906083
Ascott Singapore Raffles Place	2 Finlayson Green, Singapore 049247	Hotel	Certified	0.0128	0.013039429	0.012468812
Central Mall	1 Magazine Road, Singapore 059567	Office	Platinum	0.0129	0.011478217	0.010694893
Singapore Chinese Cultural Centre	1 Straits Boulevard, Singapore 018906	Civic, Community and Cultural Institution	Platinum	-	0.005407592	0.005586921
Chijmes	30 Victoria Street, Singapore 187996	Retail	Certified	0.0346	0.035158891	0.004620634
Tokio Marine Centre	20 Mccallum Street, Singapore 069046	Office	GoldPlus	0.0107	0.010905869	0.011243598

People's Association	9 King Georges Avenue, Singapore 208581	Office	GoldPlus	0.01	0.009899638	-
Intercontinental Singapore Robertson Quay	1 Nanson Road, Singapore 238909	Commercial Building	Certified		0.027366368	0.027838604
Singapore Flyer	30 Raffles Avenue, Singapore 039803	Commercial Building	Certified	-	0.029300832	0.03026838
Macpherson Mall	401 Macpherson Road, Singapore 368125	Mixed Development	Certified	0.0145	0.017088226	0.017491705
Singapore Pools Building	210 Middle Road, Singapore 188994	Office	GoldPlus	0.0201	0.020120418	0.020766043
Land Transport Authority	10 Sin Ming Drive, Singapore 575701	Office	Platinum	0.023	0.022880633	-
Hotel Royal @ Queens	12 Queen Street, Singapore 188553	Hotel	Gold	0.0185	0.018304815	0.017954348
158 Cecil Street	158 Cecil Street, Singapore 069545	Office	Gold	0.0188	0.018201553	0.016944837
Holiday Inn Express Singapore Clarke Quay	2 Magazine Road, Singapore 059573	Hotel	Platinum	0.0224	0.022320292	0.022760561
Nanyang Academy Of Fine Arts (NAFA) Campus 3	151 Bencoolen Street, Singapore 189656	Private College	Platinum	0.00992	0.011650988	0.011472212
30 Hill Street	30 Hill Street, Singapore 179360	Office	Gold	0.0192	0.017939967	0.016516711
Village Hotel Albert Court	180 Albert Street, Singapore 189971	Commercial Building	Platinum	-	0.018575216	0.019133999
OCBC Centre South	18 Church Street, Singapore 049479	Office	Gold	0.0348	0.035937359	0.036189647
King's Centre	390 Havelock Road, Singapore 169662	Office	Platinum	0.0202	0.018890527	0.017345205
North Bridge Centre	420 North Bridge Road, Singapore 188727	Office	Gold	0.0149	0.014949804	0.015665702

Hotel Fort Caning	11 Canning Walk, Singapore 178881	Hotel	GoldPlus	0.0382	0.036538893	-
Hotel G Singapore	200 Middle Road, Singapore 188980	Commercial Building	Certified	-	0.049369241	0.046481973
One Raffles Place	55 Market Street, Singapore 048941	Office	Gold	0.0234	0.021264028	-
Studio M Hotel	3 Nanson Road, Singapore 238910	Hotel	Gold	0.0529	0.05225335	0.056845835
National Design Centre	111 Middle Road, Singapore 188969	Office	Platinum	0.0252	0.02499913	0.027416813
Beach Centre	15 Beach Road, Singapore 189677	Office	Platinum	0.0202	0.023340722	0.021449698
Maxwell Chambers Suites	28 Maxwell Road, Singapore 069120	Commercial Building	Certified	-	-	0.016945593
Lucky Chinatown	211 New Bridge Road, Singapore 059432	Commercial Building	Gold	-	0.049846645	0.052032751
36|38 Armenian	36 Armenian Street, Singapore 179934	Office	Certified	0.0175	0.018810371	0.016415727
NTUC Trade Union House	73 Bras Basah Road, Singapore 189556	Private School	Gold	0.0456	0.042432815	0.041387248
Registry Of Marriages	7 Canning Rise, Singapore 179869	Commercial Building	Gold	-	0.016833349	0.017773551
Grace Global Raffles	137 Market Street, Singapore 048943	Office	GoldPlus	0.034	0.032803181	0.032906911
Marina At Keppel Bay	2 Keppel Bay Vista, Singapore 098382	Mixed Development	Gold	0.358	0.353	-
